# DDAVP Might Reduce the Risk of Preeclampsia in Pregnant Women with VWF Deficiency

**DOI:** 10.4274/Tjh.2012.0044

**Published:** 2013-06-05

**Authors:** Alireza Hamidian Jahromi, Mehran Karimi

**Affiliations:** 1 Louisiana State University Health Sciences Center, Department of Surgery, Shreveport, LA, United States; 2 Hematology Research Center, Shiraz University of Medical Sciences, Shiraz, Iran

**Keywords:** DDAVP, preeclampsia, Pregnancy, deficiency, Platelet

## TO THE EDITOR

Preeclampsia is the most common medical disorder of pregnancy [1]. Preeclampsia can present very early in pregnancy with hypertension and proteinuria. Preeclampsia includes a spectrum of clinical symptoms varying from subtle weight gain and swelling of the face and extremities to severe headache, abdominal pain, nausea, vomiting, and visual impairment. Endothelial cell changes along with widespread fibrin deposition in the microvasculature are parts of the proposed pathophysiological changes in this medical condition [1]. Presence of an impaired fibrinolysis pathway along with the overactive coagulation cascade present in the patients with severe preeclampsia can lead to multiple organ dysfunctions [1].

Women with early-onset preeclampsia, i.e. presentation earlier than 34 weeks gestational age, constitute a subgroup of patients with more severe and more common hematologic abnormalities [1]. The Paris Collaborative Group [2] performed a metaanalysis of 31 randomized trials of preeclampsia primary prevention, which included more than 32,000 pregnant women, and proposed that administration of antiplatelet agents during pregnancy was associated with “moderate but consistent reductions in the relative risk of preeclampsia” [2]. All of the above evidence supports the theory that platelet–endothelial interaction can have a central role in the pathogenesis of preeclampsia.

Von Willebrand Factor (VWF) is a polymeric plasma glycoprotein comprising multiple 250-kDa subunits that facilitates platelet adhesion to the vessel wall by linking platelet membrane receptors to the subendothelium and is the cornerstone of platelet–endothelial interaction. It also serves as the plasma carrier for Factor VIII and actually stabilizes the molecule. 

VWF is synthesized and released from the endothelial cells and megakaryocytes into the circulation [3]. Desmopressin (DDAVP) induces release of stored factor VIII and VWF into the circulation. Due to this effect, DDAVP is a known treatment for mild VWF and factor VIII deficiency. It has been suggested that DDAVP use during pregnancy is safe for both mother and child [4]. In a review of the literature on the safety of DDAVP during pregnancy, Ray found no evidence to support the idea that this medication might interact with oxytocin, cause early delivery, or have neonatal adverse effects [4]. DDAVP (0.3 µg/kg) was used in a preeclamptic patient with prolonged bleeding time in an attempt to correct her bleeding tendency and prepare her for epidural anesthesia [5]. It might be the case that DDAVP usage in pregnancy has a protective effect against the development of preeclampsia in women with VWF deficiency, as well. According to this hypothesis, DDAVP can be used in a single IV dose of 0.33 µg/kg between 28 and 34 weeks of gestational age, when risk of preeclampsia is high, with blood pressure being monitored while the DDAVP is used. It is safe during pregnancy and a randomized clinical trial is proposed to confirm this hypothesis.

On the other hand, VWF deficiency could be a risk factor for preeclampsia. Given this fact, it might be possible that women with VWF deficiency will have increased risk of developing preeclampsia. Thus, in the case of preeclampsia associated with bleeding symptoms, VWF deficiency should be taken into account. DDAVP can be considered in such cases as an alternative treatment.

## References

[1] Heilmann L, Rath W, Pollow K (2007). Hemostatic abnormalities in patients with severe preeclampsia. Clin Appl Thromb Hemost.

[2] Askie LM, Duley L, Henderson-Smart DJ (2007). Antiplatelet agents for prevention of pre-eclampsia: a meta-analysis of individual patient data. Lancet.

[3] Cohan N, Karimi M (2011). Diagnosis and management of von Willebrand disease in Iran. Semin Thromb Hemost.

[4] Ray JG (1998). DDAVP use during pregnancy: an analysis of its safety for mother and child. Obstet Gynecol Surv.

[5] Fragneto RY, Datta S (1995). Epidural analgesia in a preeclamptic parturient after normalization of a prolonged bleeding time with DDAVP. Reg Anesth.

